# Supplementary material for: Scarlet Flax Linum grandiflorum (L.) In Vitro Cultures as a New Source of Antioxidant and Anti-Inflammatory Lignans
Source: Molecules. 2021 Jul 27;26(15):4511. doi: 10.3390/molecules26154511 (PMC8348589; doi:10.3390/molecules26154511)
Supplement: Supplementary file 1 [file molecules-26-04511-s001.zip › molecules-1285028-supplementary.pdf]

Supplementary Materials

Scarlet Flax *Linum grandiflorum* (L.) *in vitro* Cultures as a New Source of Antioxidant and Anti-inflammatory Lignans

Bushra Asad [1], Taimoor Khan [1], Faiza Zareen Gul [1], Muhammad Asad Ullah [1], Samantha Drouet [2], Sara Mikac [2], Laurine Garros [2], Manon Ferrier [3], Shankhamala Bose [3], Thibaut Munsch [3], Duangjai Tungmunnithum [2,4], Arnaud Lanoue [3], Nathalie Giglioli-Guivarc'h [3], Christophe Hano [2,*], Bilal Haider Abbasi [1,*]

Figure S1: Total phenolic contents in the hypocotyl- and cotyledon-derived callus cultures of *L. grandiflorum* grown on PGRs (NAA and/or TDZ) treatments.

Figure S2: Typical HPLC chromatogram of *L. grandiflorum* callus extract showing the presence of SECO, LARI and DCA.

Table S1: PGRs hormonal applications (mg/L) and their effect on various parameters of callus cultures of *Linum grandiflorum*.DG; Dark green, LG: Light green, YG; Yellowish green, C; Compact, F; Friable.

Table S2: Pearson correlation coefficients showing the relation between the biomass (expressed in DW basis) and the main phytochemicals of *in vitro* cultures of *L. grandiflorum* (L.).

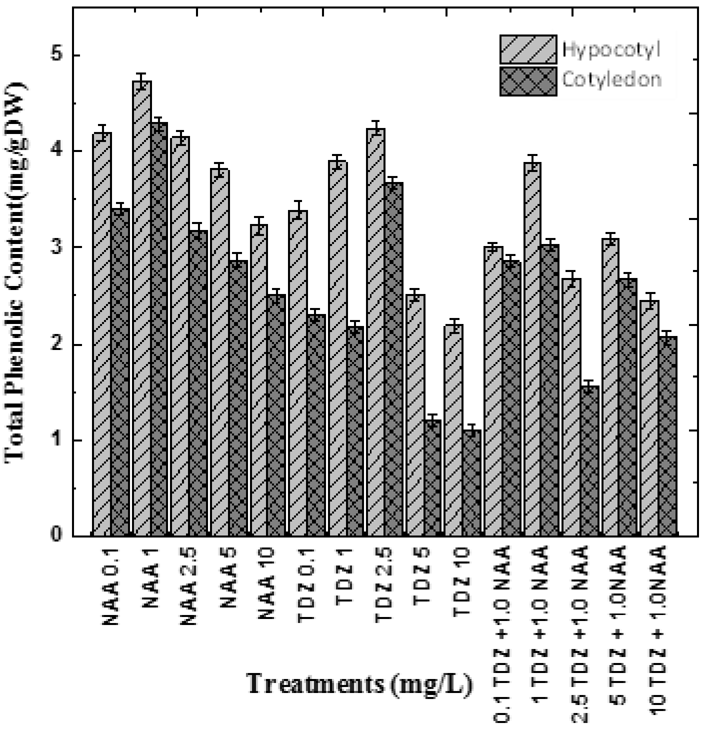

Figure S1: Total phenolic contents in the hypocotyl- and cotyledon-derived callus cultures of *L. grandiflorum* grown on PGRs (NAA and/or TDZ) treatments.

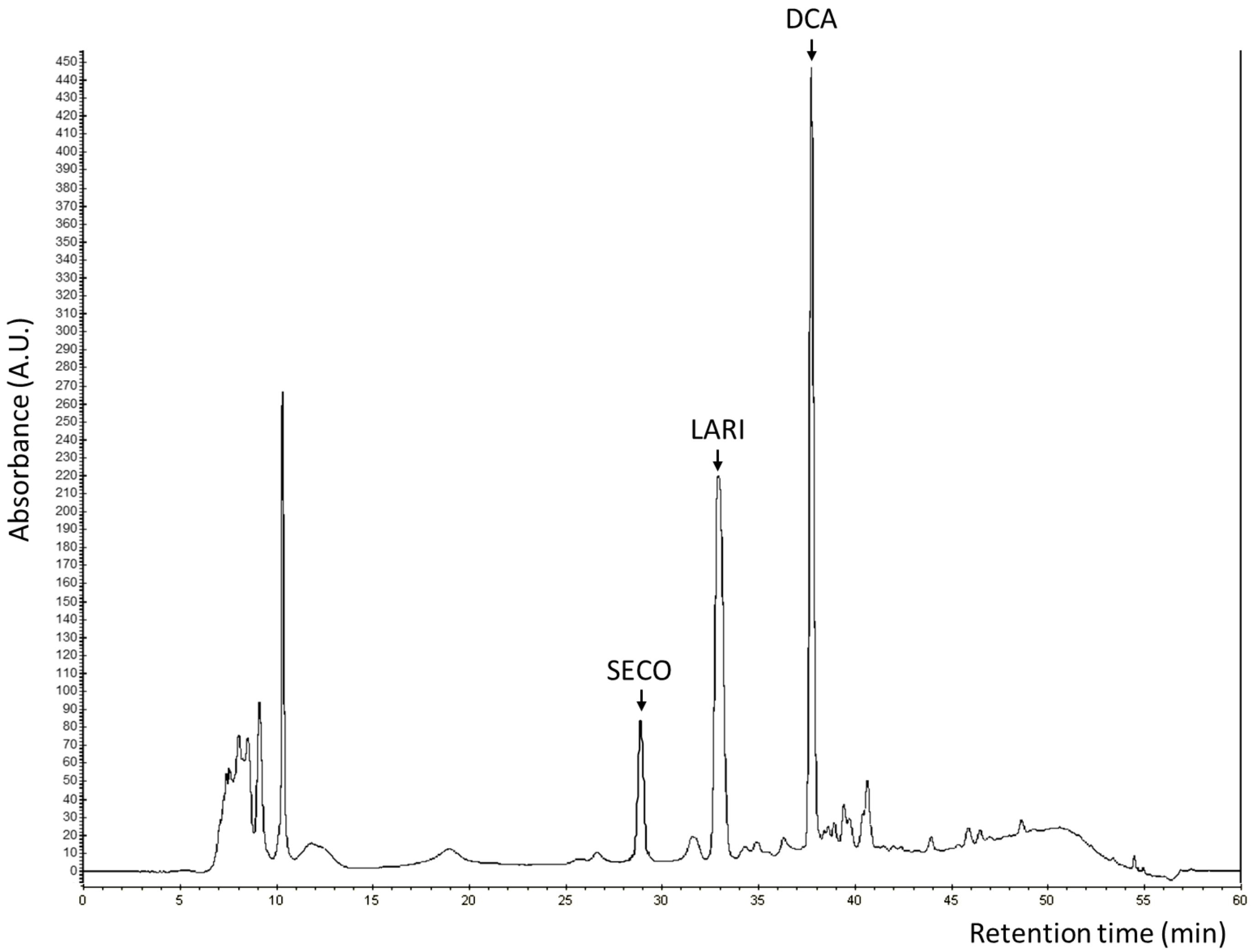

Figure S2: Typical HPLC chromatogram of *L. grandiflorum* callus extract showing the presence of SECO, LARI and DCA.

Table S1: PGRs hormonal applications (mg/L) and their effect on various parameters of callus cultures of *Linum grandiflorum.* DG; Dark green, LG: Light green, YG; Yellowish green, C; Compact, F; Friable.

S. No	PGR's Treatment mg/L	Stem Explant				Leaf Explant			
		Callus Initiation (day)	Callus Induction Frequency	Callus color	Callus texture	Callus Initiation (day)	Callus Induction frequency	Callus color	Callus texture
1	MS+0.1NAA	14	89	DG	F	15	83	DG	C
2	MS+1.0NAA	14	95	DG	F	15	87	DG	C
3	MS+2.5NAA	14	92	DG	F	15	85	DG	C
4	MS+5.0NAA	14	77	DG	F	15	77	DG	C
5	MS+10 NAA	14	81	DG	F	15	70	DG	C
6	MS+0.1TDZ	17	51	G	C	18	48	LG	C
7	MS+1.0TDZ	17	49	G	C	18	44	LG	C
8	MS+2.5TDZ	17	66	G	C	18	55	LG	C
9	MS+5.0TDZ	17	33	G	C	81	31	LG	C
10	MS+1.0TDZ	17	48	G	C	18	41	LG	C
11	MS+0.1TDZ +1.0NAA	15	55	LG	F	16	50	YG	C
12	MS+1.0TDZ +1.0NAA	15	67	LG	F	16	61	YG	C
13	MS+2.5TDZ +1.0NAA	15	51	LG	C	16	47	YG	C
14	MS+5.0TDZ +1.0NAA	15	60	YG	C	16	56	YG	C
15	MS+10TDZ +0.1NAA	15	49	YG	C	16	41	YG	C

Table S2: Pearson correlation coefficients showing the relation between the biomass (expressed in DW basis) and the main phytochemicals of *in vitro* cultures of *L. grandiflorum* (L.)

	SECO	LARI	DCA
Biomass	0.323 ns	0.643 ***	0.326 ns

Significance level: *** $p < 0.001$, ns: not significant.
